# *Capnocytophaga canimorsus:* A Human Pathogen Feeding at the Surface of Epithelial Cells and Phagocytes

**DOI:** 10.1371/journal.ppat.1000164

**Published:** 2008-09-26

**Authors:** Manuela Mally, Hwain Shin, Cécile Paroz, Regine Landmann, Guy R. Cornelis

**Affiliations:** 1 Infection Biology, Biozentrum, University of Basel, Klingelbergstrasse, Basel, Switzerland; 2 Infection Biology, Department of Research, University Hospital Basel, Hebelstrasse, Basel, Switzerland; Dartmouth Medical School, United States of America

## Abstract

*Capnocytophaga canimorsus*, a commensal bacterium of the canine oral flora, has been repeatedly isolated since 1976 from severe human infections transmitted by dog bites. Here, we show that *C. canimorsus* exhibits robust growth when it is in direct contact with mammalian cells, including phagocytes. This property was found to be dependent on a surface-exposed sialidase allowing *C. canimorsus* to utilize internal aminosugars of glycan chains from host cell glycoproteins. Although sialidase probably evolved to sustain commensalism, by releasing carbohydrates from mucosal surfaces, it also contributed to bacterial persistence in a murine infection model: the wild type, but not the sialidase-deficient mutant, grew and persisted, both when infected singly or in competition. This study reveals an example of pathogenic bacteria feeding on mammalian cells, including phagocytes by deglycosylation of host glycans, and it illustrates how the adaptation of a commensal to its ecological niche in the host, here the dog's oral cavity, contributes to being a potential pathogen.

## Author Summary

*Capnocytophaga canimorsus* is a commensal bacterium of dogs/cats oral flora, which causes rare but severe infections in humans that have been bitten or simply licked by a dog/cat. Fulminant septicemia and peripheral gangrene are most common symptoms. Although splenectomy has been identified as a predisposing factor, some 40% of the patients have no immunosuppression history. *C. canimorsus* belongs to the phylum *Bacteroidetes,* which includes many commensals of the human gut flora but few pathogens. *C. canimorsus* has been shown previously to be immunosuppressive and to resist phagocytosis by macrophages. Here, we show that this bacterium feeds on surface-exposed glycoproteins from cultured mammalian cells. This property, which was found to depend on a bacterial surface-exposed sialidase, suggests that in its natural niche—the dog's oral cavity—*C. canimorsus* may feed on the dog's mucosal cells. Moreover, we found that *C. canimorsus* also feeds on phagocytes and that sialidase contributes to persistence and virulence in a mouse infection model. Thus, by adapting to its ecological niche, *C. canimorsus* also developed the potential to persist within the tissues of an infected host. This observation nicely illustrates how commensalism and pathogenesis are two faces of the same coin.

## Introduction

*Capnocytophaga canimorsus* (formerly Centers for Disease Control group DF-2) has been rarely but regularly isolated from dog or cat bite infections since its discovery in 1976 [1],[2]. *C. canimorsus* is a thin Gram-negative rod, found in the normal oral flora of dogs and cats. Clinical infections by *C. canimorsus* generally appear as fulminant septicemia, peripheral gangrene or meningitis [3],[4]. Splenectomy, alcohol abuse and immunosuppression have been associated with a number of cases, but more than 40% of the patients have no obvious risk factor [5]. *Capnocytophaga* belongs to the family of *Flavobacteriaceae* in the phylum of *Bacteroidetes,* which is taxonomically remote from Proteobacteria. The family of *Bacteroidaceae* contains many commensals of the mammalian intestinal system such as *Bacteroides thetaiotaomicron* and *Bacteroides fragilis* [6]. The family of *Flavobacteriaceae* includes a variety of environmental and marine bacteria, among which *Flavobacterium johnsoniae* is a common soil and freshwater bacterium studied for its gliding motility [7]. There are only a few examples of pathogenic bacteria belonging to this family. These are *Flavobacterium psychrophilum,* the causative agent of cold water disease in salmonid fish [8], *Ornithobacterium rhinotracheale,* a bacterial pathogen known for causing respiratory disease in poultry [9], and *Riemerella anatipestifer* which causes “duckling disease” in waterfowl and turkeys [10],[11]. The genus *Capnocytophaga* includes seven species found in normal human oral flora and *C. canimorsus* found in the normal flora of dogs and cats. More than 160 cases of *C. canimorsus* infections have been reported so far [12] but very few studies have addressed the molecular mechanisms of *C. canimorsus* pathogenesis. Recently, we showed that *C. canimorsus 5 (Cc5*) resists phagocytosis by macrophage cell line [13],[14]. We also showed that although *C. canimorsus* does not affect the viability of murine or human macrophages, it does not elicit proinflammatory cytokines and it even blocks the proinflammatory response to the LPS from enterobacteria [13]. In the course of such experiments, *Cc5* exhibited robust growth, although it is usually considered fastidious for growth. In the present study, we show that a surface-localized sialidase plays a key role in initiating an extensive deglycosylation process of host cell glycan structures and that this feeding mechanism serves as a basis for growth and persistence of *C. canimorsus in vivo*.

## Results

### Growth of *C. canimorsus 5* requires direct contact with cells

**Figure 1:**
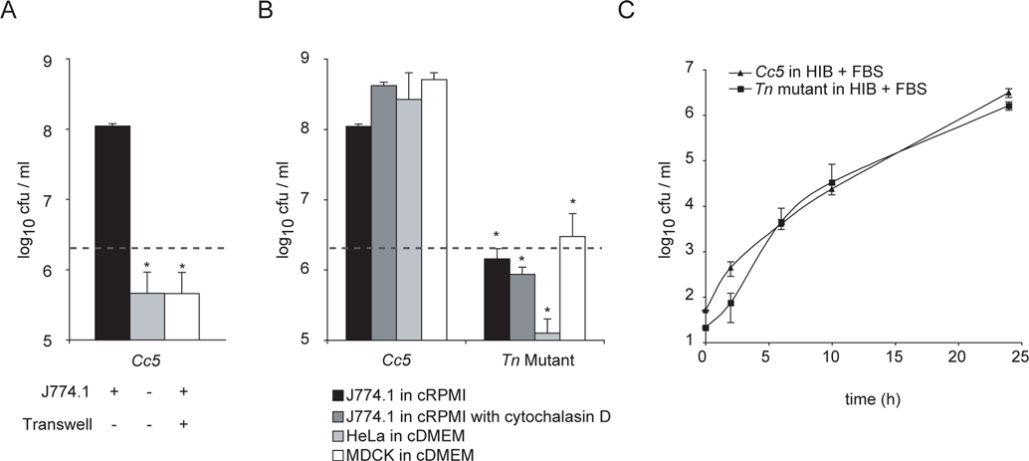
Growth of *C. canimorsus 5* is dependent on cell contact. (A) Viable counts of 2×10^6^ *Cc5* after 24 h in presence of J774.1 macrophages in RPMI supplemented with 10% FBS (moi = 20) (black) or in RPMI with FBS without cells (grey) and in a transwell system preventing physical contact between bacteria and macrophages in RPMI with FBS (white). (B) Viable counts of *Cc5* and Tn mutant after 24 h culture with macrophages in RPMI and FBS (black), with macrophages in RPMI and FBS in addition of cytochalasin D (grey), with HeLa cells (light grey) and MDCK cells in DMEM and FBS (white). The grey dotted line represents the bacterial number inoculated. The difference is statistically significant between *Cc5* and Tn mutant (2-tailed unpaired Student's t test p<0.05) in 3 or more experiments. (C) Growth curve of wt *Cc5* (triangles) and Tn mutant (squares) in heart infusion broth (HIB) supplemented with 10% FBS, represented as the mean of 3 or more experiments with the error bars showing the s.d.

When inoculated at a multiplicity of infection (moi) of 20 to J774.1 murine macrophages cultured in complete RPMI (cRPMI), which includes 10% fetal bovine serum (FBS), *Cc5* multiplied about 100-fold during the 24 h of infection (Fig. 1A). This observation could be repeated with non-phagocytic human epithelial HeLa cells and with canine epithelial MDCK kidney cells (Fig. 1B). Surprisingly, growth was abolished when J774.1 macrophages were omitted and moreover, *Cc5* was unable to grow in a transwell system, indicating that direct contact is required for bacterial growth (Fig. 1B). This implies that *Cc5* may take advantage of some nutrient that is present on the host cell surface. Notably, *Cc5* did not adhere tightly to cells and was not internalized (data not shown). We generated a transposon (Tn) mutant library using Tn*4351* from *B. fragilis* [15],[16] and isolated a clone that was unable to grow in the presence of J774.1 cells, but grew normally on blood agar plates. Wild type (wt) and mutant bacteria grew equally well in serum enriched heart infusion medium (Fig. 1C). Impaired growth of this Tn mutant was not due to an increased phagocytic uptake by J774.1 since addition of cytochalasin D had little effect on bacterial growth (Fig. 1B).

### Surface-localized sialidase is required for the growth of *Cc5* in contact with cells

**Figure 2:**
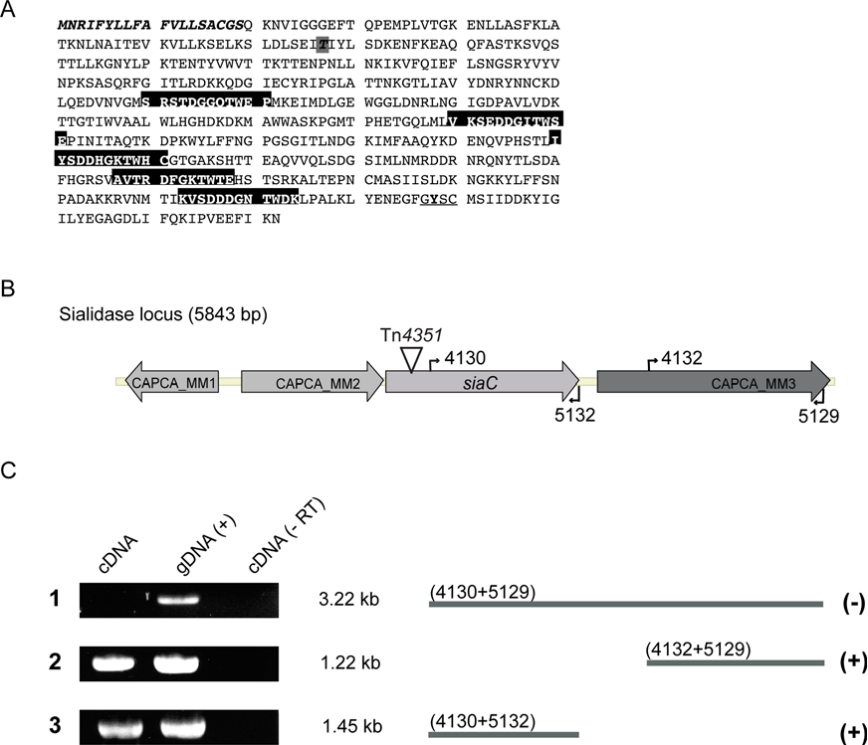
Identification of the Tn integration site and analysis of mRNA present in wt *C. canimorsus* 5. (A) Amino acid sequence of the *C. canimorsus* sialidase showing the signal peptide (italics) and the BNR/asp repeats (Ser/Thr-X-Asp-X-Gly-X-Thr-Trp/Phe) of bacterial sialidases (boxed). Domain predictions were analyzed by InterProScan [42]. The residues conserved in sialidases at the C-terminus are underlined and the tyrosine 488 is bold [43]. The Tn*4351* integration site in SiaC at amino acid 77 is indicated, boxed in grey and bold. (B) Genetic locus of the sialidase gene (*siaC*) including its upstream genes, *gntR*-like gene (CAPCA_MM1) and putative N-acyl-glucosamine epimerase encoding gene (CAPCA_MM2); and downstream coding sequence (CAPCA_MM3). (C) Reverse transcription performed on total RNA with specific primers (5129 or 5132) followed by PCR to identify transcripts present in wt *Cc5* (cDNA). PCR reactions were also performed using genomic DNA (gDNA) as template instead of cDNA as a positive control. As a control, reverse transcription was performed without reverse transcriptase in a parallel assay and used as template for the subsequent PCR reaction (-RT).

The transposon inserted at codon 77 within a gene encoding a protein with similarity to bacterial sialidase, glycosylhydrolase that cleaves terminal sialic acid from glycoconjugates (Fig. 2A). The mutated gene, designated *siaC,* was found to be located downstream of genes encoding a predicted transcriptional regulator and a putative N-acyl-glucosamine epimerase (accession number: EU329392). The first gene downstream was found to start 148 bp further from the *siaC* stop codon (Fig. 2B). To exclude any polar effects of the Tn integration, we tested whether the downstream gene was transcriptionally linked to *siaC*. Total RNA was isolated from wt *Cc5,* reverse transcribed using two different primers annealing either at the end of *siaC* (5132) or at the end of the downstream gene (5129) and the cDNA was amplified by PCR. As shown in Fig 2C, even though transcripts were present for both genes separately, no transcript spanned *siaC* and the downstream gene. This result indicates that *siaC* is not transcriptionally linked to the downstream gene.

**Figure 3:**
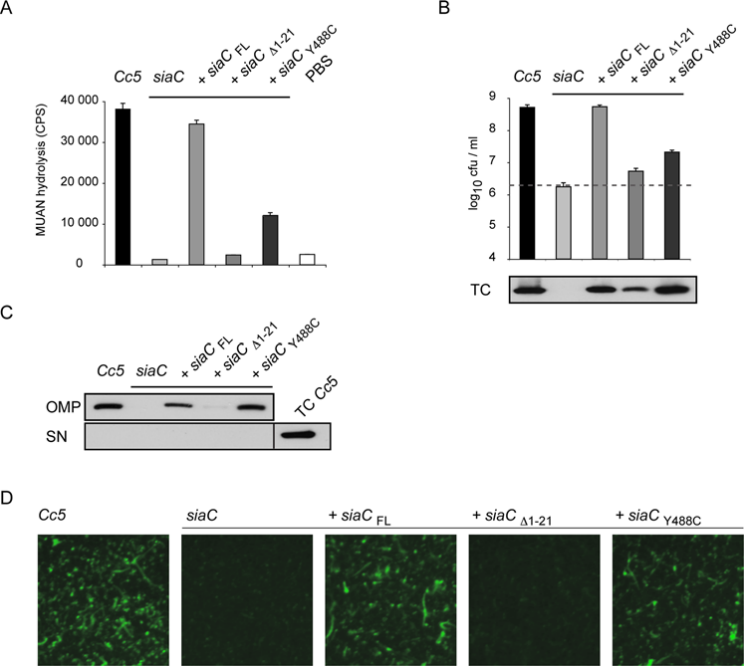
Surface localized sialidase is required for growth. (A) Sialidase activity of intact bacteria, measured with the substrate MUAN as the mean of triplicate measurements and s.d. of a representative experiment. (B) Viable counts after challenge with 2×10^6^ *Cc5* (black), *siaC* (light grey) or *siaC* complemented with plasmids containing *siaC*_FL_, *siaC*_Δ1–21_ and *siaC*_Y488C_ after 24 h in presence of J774.1 with the grey dotted line indicating the bacterial number inoculated. Sialidase was detected by immunoblotting with a polyclonal antibody against SiaC in total cells (TC). (C) Outer membrane protein fractions (OMP), cell free supernatants (SN) of the J774.1 cultures shown in (B) including as control TC of *Cc5* were analyzed by immunoblotting for the presence of SiaC. (D) Surface localization of SiaC was tested by immunofluorescence on paraformaldehyde fixed but not permeabilized bacteria using anti-SiaC followed by anti- rabbit IgG conjugated to FITC.

While intact *Cc5* bacteria cleaved 2′-(4-Methylumbelliferyl)-α-D-N-acetylneuraminic acid (MUAN), the Tn mutant could not, indicating that the mutated gene does indeed encode a sialidase (Fig. 3A). We engineered an expression shuttle vector by taking advantage of a cryptic plasmid isolated from another strain of *C. canimorsus* and the promoter of an insertion sequence from *B. fragilis* [16]. We constructed plasmids encoding full length (FL) SiaC, a variant deprived of the 21 N-terminal residues, predicted to be a signal peptide (Δ1–21), and a catalytic mutant (Y488C). Sialidase activity (Fig. 3A) and growth in the presence of J774.1 cells (Fig. 3B) was restored by introducing *in trans siaC*_FL_, but not *siaC*_Δ1–21_. Sialidase activity was not restored to wt levels by *siaC*_Y488C_, but it was still significant (Fig. 3A), suggesting that this residual activity might account for elevated growth in comparison to the *siaC* mutant (Fig. 3B). Using a sarcosyl extraction method, SiaC_FL_ and SiaC_Y488C_ were found to be associated with the outer membrane (Fig. 3C), whereas SiaC_Δ1–21_ was only detected in total cells (Fig. 3B). Indirect immunofluorescence using polyclonal anti-SiaC serum on paraformaldehyde fixed but unpermeabilized bacteria confirmed that SiaC is exposed on the bacterial surface unless the signal peptide is removed (Fig. 3D). Although it is surface exposed, no SiaC could be detected in the supernatant of infected J774.1 cultures, indicating that it is tightly associated with the outer membrane (Fig. 3C). Hence, surface-localized sialidase is required for growth of *Cc5* at the expense of mammalian cells.

### Growth is sustained by N-acetyl glucosamine (GlcNAc) and N-acetyl galactosamine (GalNAc) but not by sialic acids

**Figure 4:**
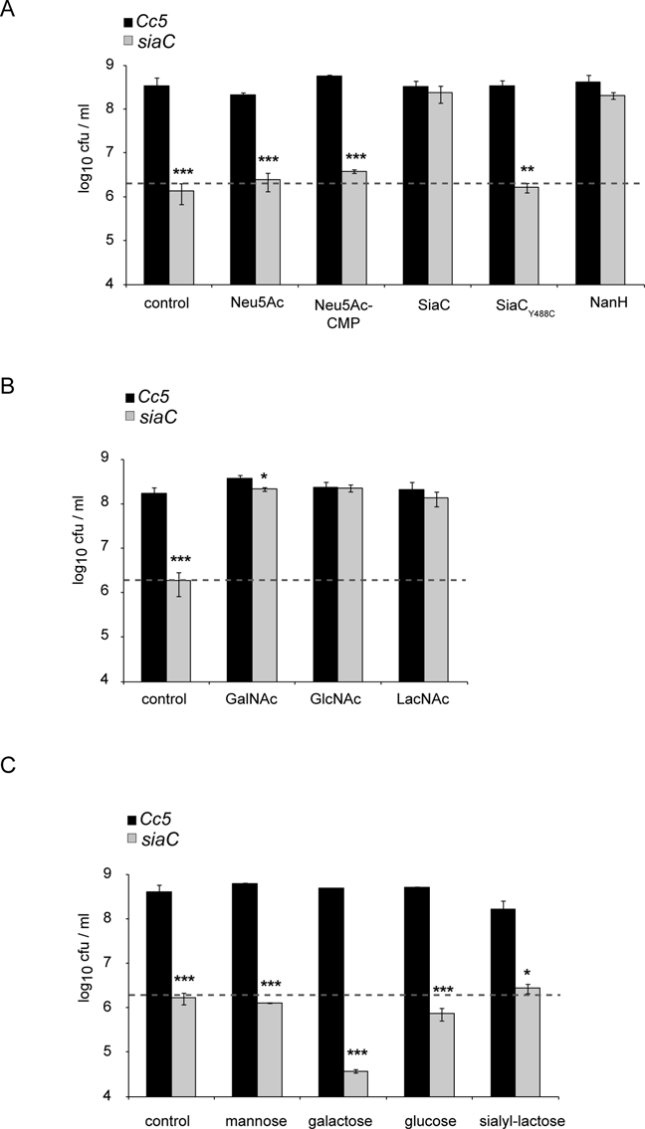
Aminosugars but not sialic acids sustain growth of *C. canimorsus.* Viable counts after challenge with 2×10^6^ wt *Cc5* (black) or *siaC* (grey) grown for 24 h with J774.1 in cRPMI (control) or in the same condition with the addition of Neu5Ac, Neu5Ac- CMP, 12.5 ng/ml enzyme SiaC_FL_, SiaC_Y488C_ or NanH from *C. perfringens* (A) or with the addition of GalNAc, GlcNAc or LacNAc (B) or with the addition of mannose, galactose, glucose or sialyl-lactose (C). Mean values from 3 or more experiments and s.d. are shown including statistical difference between wt *Cc5* and *siaC* with * p<0.05, ** p<0.01 and *** p<0.001 for each pair of columns (2-tailed unpaired Student's t test). The grey dotted line indicates the bacterial number inoculated.

Since sialidases cleave terminal sialic acid from glycoconjugates, we first tested whether the addition of sialic acids could restore growth of *siaC*. Addition of neither sialic acid (Neu5Ac, N-Acetyl-2,3-dehydro-2-deoxyneuraminic acid) nor its activated form (CMP-Neu5Ac, Cytidine-5′-monophospho-N-acetylneuraminic acid) restored growth of *siaC* in presence of J774.1. In contrast, growth could be restored by the addition of purified recombinant SiaC or neuraminidase/sialidase NanH from *Clostridium perfringens* to the culture medium, but not by the addition of the catalytically inactive SiaC_Y488C_ (Fig. 4A). This suggested that removal of terminal sialic acids from glycoconjugates is required to make other carbohydrates accessible. Indeed, N-acetyl glucosamine (GlcNAc) and N-acetyl galactosamine (GalNAc), common carbohydrate moieties of glycoconjugates, allowed growth of *siaC* in the presence of macrophages (Fig. 4B). Notably, addition of glucose (Glc), galactose (Gal), mannose (Man) or sialyl-lactose (N-acetylneuraminosyl-D-lactose) could not restore growth of *siaC* bacteria (Fig. 4C). As galactose (Gal) is a common sugar preceding GlcNAc in glycan molecules, we next tested addition of N-acetyl lactosamine (LacNAc), a disaccharide consisting of β-D-Gal β(1→4) GlcNAc. LacNAc also restored the growth defect of *siaC* indicating the presence of an active β-galactosidase releasing monosaccharides Gal and GlcNAc in wt and *siaC Cc5* (Fig. 4B).

### Sialidase desialylates macrophage and epithelial cell surfaces

**Figure 5:**
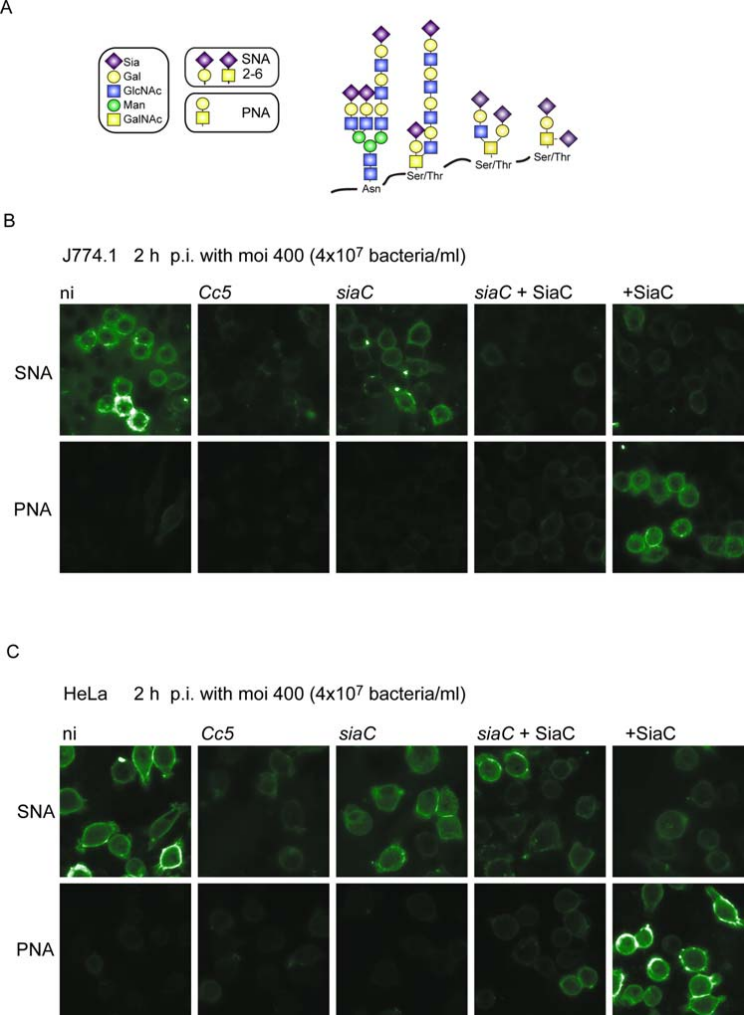
*C. canimorsus* desialylates macrophage and epithelial cell surfaces. (A) The targets of the lectins used in this study are schematically represented (adapted from [44]). Surface carbohydrates of J774.1 macrophages (B) or HeLa epithelial cells (C) were analyzed by lectin binding after 2 h of infection with 4×10^7^ wt (*Cc5*) or *siaC* bacteria. Cells were fixed with paraformaldehyde and incubated for 1 h with lectin SNA, which recognizes terminal sialic acids (2- 6 or 2- 3) linked to Gal or to GalNAc or PNA that binds to the disaccharide Gal 1–3 GalNAc only after removal of terminal sialic acids. SiaC was added to cells alone or with *siaC* bacteria at 100 ng/ml. Biotinylated lectins were visualized by FITC conjugated streptavidin.

J774.1 macrophages were incubated with either wt or *siaC* bacteria and thereafter analyzed for lectin binding to investigate desialyation process on the macrophage cell surface. We used *Sambucus nigra* agglutinin (SNA), which recognizes terminal sialic acids (2- 6 or 2- 3) linked to Gal or to GalNAc, and peanut agglutinin (PNA), a lectin specific for Gal (β 1–3) GalNAc, a disaccharide often forming the core unit of O-linked glycoconjugates (Fig. 5A). As shown in Fig. 5B, wt bacteria greatly reduced the amount of sialic acids (SNA panel) and Gal (β 1–3) GalNAc (PNA panel) at the cell surface, while *siaC* bacteria had no effect on glycans masked by sialic acids. When cells were treated simultaneously with purified SiaC and *siaC* bacteria, neither sialic acid nor Gal (β 1–3) GalNAc were detected, indicating that *siaC* bacteria are still proficient in extensive deglycosylation of exposed glycans chains. The same deglycosylation of cell surfaces was observed when HeLa epithelial cells were used (Fig. 5C).

### Sialidase inhibitor N-Acetyl-2,3-dehydro-2-deoxyneuraminic acid (Neu5Ac2en) can inhibit growth of *Cc5*

**Figure 6:**
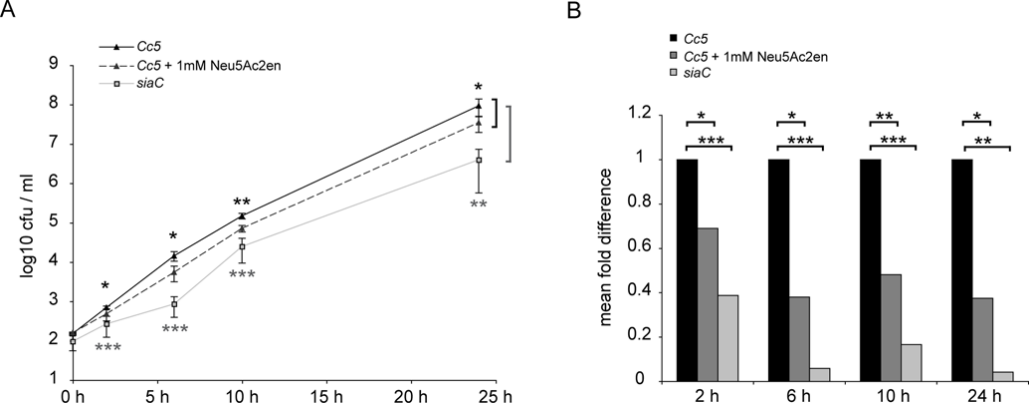
The sialidase inhibitor Neu5Ac2en decreases growth of wt *C. canimorsus 5* in presence of macrophages. (A) Viable counts of approximately 150 bacteria grown in cRPMI in the presence of J774.1 cells for 2, 6, 10 and 24 h: *siaC* bacteria (light grey); wt *Cc5* bacteria with 1mM Neu5Ac2en (grey, dotted line); wt *Cc5* in the absence of inhibitor (black). Mean values from 4 experiments and s.d. are shown including statistical difference between *Cc5* and *siaC* in grey or between *Cc5* and *Cc5* treated with Neu5Ac2en in black with * p<0.05, ** p<0.01 and *** p<0.001 (2-tailed unpaired Student's t test). (B) Data from viable counts (mean) shown in (A) is represented as the fold difference compared to wt *Cc5.* The statistical difference is depicted from (a) with * p<0.05, ** p<0.01 and *** p<0.001.

As bacterial and viral sialidases can share common ASP boxes that interact with sialic acid, we postulated that common sialidase inhibitor might have sufficient specificity for the active site in SiaC to inhibit growth of *Cc5* wt bacteria in presence of cells. We tested the anhydro sialate derivative Neu5Ac2en, which is known to inhibit many viral and bacterial neuraminidases [17],[18]. Approximately 150 cfu/ml of wt *Cc5* were inoculated to a culture of J774.1 macrophages in the presence of 1mM Neu5Ac2en and growth was monitored after 2, 6, 10 and 24 h (Fig. 6A). Between 2 and 24 h post infection, counts of wt *Cc5* were significantly reduced to values close to the *siaC* mutant (Fig. 6B). These data indicate that Neu5Ac2en has affinity for the active site of SiaC and restricts the growth of *Cc5* in the presence of J774.1 cells.

### *Cc5* but not *siaC* is able to persist in murine tissue cages

**Figure 7:**
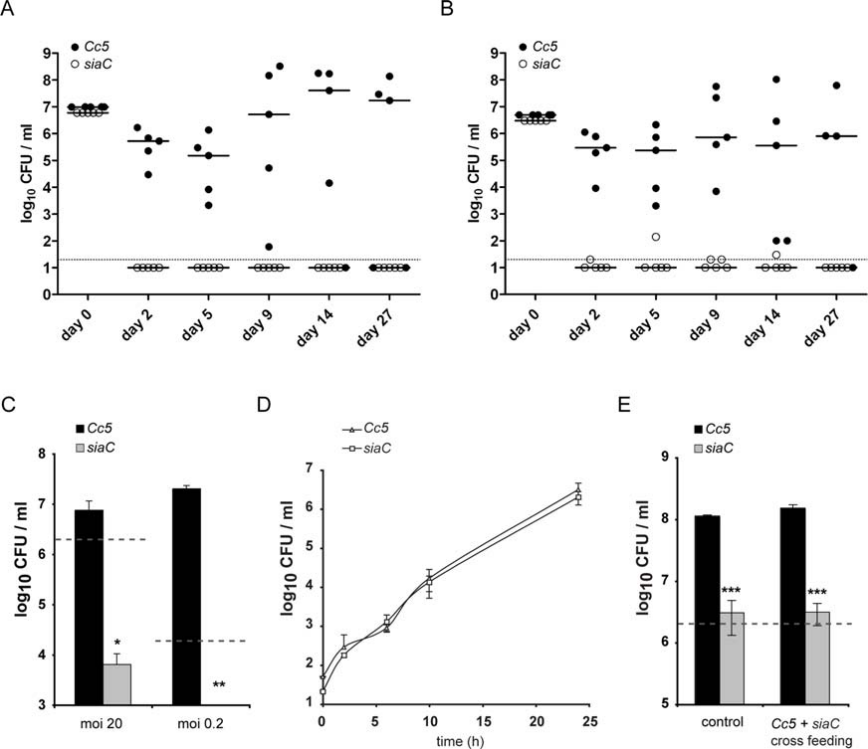
The sialidase mutant is hypo-virulent in a tissue cage mouse infection model. Tissue cages were implanted in C57BL/6 mice and infected with 10^7^ *Cc5* wt and *siaC* bacteria (n = 5) singly (A) or in competition (B). Bacteria were counted in tissue cage fluid (TCF) during 27 days (*Cc5 = *black circles; *siaC* = open circles). Individual values are shown; horizontal lines indicate the median value of each group. The black dotted line is the detection limit of 20 bacteria per ml TCF. (A) Cfu numbers between groups were significantly different on days 2, 5 and 9 with p<0.01 and on days 14 and 27 with p<0.05 (Mann Whitney test). (B) 10^7^ cfu *Cc5* and erythromycin resistant *siaC* were inoculated at a 1∶1 ratio. Bacterial numbers were analyzed for 27 days (n = 5). Viable counts between wt and *siaC* were significantly different on day 2, 5 and 9 with p<0.01 and on day 14 with p<0.05. (C) *Ex vivo* isolated leukocytes were resuspended in serum free RPMI and inoculated at a moi of 20 (2×10^6^ bacteria) or 0.2 (2×10^4^ bacteria) indicated with grey dotted lines and bacterial viable count was monitored after 24 h. Values represent the mean using TCF cells from 3 uninfected mice. TCF leukocytes consist of 68% +/− 4.8% polymorphonuclear neutrophils (PMNs), 18% +/− 3.2% monocytes and 9.1% +/− 3.7% macrophages. Wt and *siaC* numbers were significantly different with p<0.05 (*) and p<0.001 (**) using 2-tailed unpaired student's t test. (D) *In vitro*, *Cc5* and *siaC* were tested in heart infusion broth with FBS inoculated at a 1∶1 ratio with approximately 100 bacteria total and bacterial growth was monitored for 2, 6, 10 and 24 h. (E) Viable counts after challenge with 2×10^6^ (grey dotted line) *Cc5* (black) or *siaC* (grey) grown for 24 h with J774.1 in cRPMI singly (control) or at a 1∶1 ratio (cross-feeding).

To test whether sialidase could play a role during *C. canimorsus* systemic infection, we selected a murine tissue cage infection model [19]. Around 10^7^ cfu of wt or *siaC Cc5* bacteria were injected directly into Teflon cages, which had been subcutaneously implanted in C57BL/6 mice. Colony forming units (cfu) counts of wt decreased on day 2 and 5. However, on day 9 they increased by 1 to 3 logs in 4 out of 5 mice, and were able to persist in 3 of 5 mice after 27 days post infection with more than 10^7^ bacteria per ml fluid. The *siaC* bacteria were undetectable after the second day in 5 out of 5 infected mice (Fig. 7A). After infection, the total number of leukocytes in tissue cage fluid (1.8×10^4^ +/− 1.3×10^4^ leukocytes/µl, mean +/− standard deviation, s.d.) did not significantly increase and was not related to the bacterial load, suggesting that *Cc5* infection did not lead to strong leukocyte recruitment. This was in agreement with the suppression of inflammation, which we previously reported [13]. In mixed infections, the competitive index of *siaC* bacteria was 9.7×10^−4^, 5.8×10^−7^ and 4.7×10^−7^ on day 5, 9 and 14, respectively. As observed during infection with wt *Cc5* alone, 3 mice out of 5 that were infected by both strains developed a persistent infection (Fig. 7B).

The fluid from uninfected control cages was collected and the leukocytes and liquid were tested separately for their capacity to sustain growth of *Cc5.* Interestingly, wt *Cc5* did not grow in the presence of the cell-free liquid (data not shown) but they grew in presence of leukocytes whereas *siaC* bacteria did not (Fig. 7C). Both strains grew equally well in heart infusion broth supplemented with 10% FBS, indicating a similar fitness *in vitro* (Fig. 1C). Mixed cultures in heart infusion broth supplemented with 10% FBS showed comparable growth of wt and *siaC* bacteria. Both strains reached 10^6^ cfu/ml after 24 h (Fig. 7D).

Our data from mixed infection in mice suggest that there is no cross-feeding of nutrients between wt and *siaC Cc5* (Fig. 7B). We thus tested whether there would be cross-feeding between wt and *siaC* bacteria when inoculated to J774.1 cultures. When wt and *siaC* were inoculated together at 1∶1 ratio to J774.1 cells, wt *Cc5* reached 10^8^ cfu/ml while *siaC* bacteria only reached 3×10^6^ cfu/ml 24 h post infection (Fig. 7E).

Taken together, these results demonstrate that SiaC plays an essential role in allowing persistence of wt *Cc5* in this tissue cage model and that clearance of *siaC* bacteria is not due to a growth defect per se but to an altered interaction of the mutant with the host. Since sialidase is surface-exposed, one could consider the possibility that it alters the susceptibility to complement. Hence, we checked the susceptibility of wt and *siaC Cc5* to mouse complement and found no difference (data not shown). It is also very unlikely that *siaC* bacteria have an increased sensitivity to killing by mouse leukocytes. Indeed, we tested phagocytosis and killing by human polymorphonuclear leukocytes and found no difference between wt and *siaC* bacteria (Manuscript in preparation). Hence, we conclude that the role of sialidase in infected mice is essentially nutritional.

## Discussion

Sialic acids are a family of nine carbon acid sugars among which Neu5Ac is one of the most widespread variants. Sialic acids are predominantly found at the terminal position of cell-surface and secreted eukaryotic glycan structures and are involved in many physiological processes including binding to microbes and down-regulation of innate immunity [20]–[22]. Therefore it is not surprising that sialic acids play a role in a variety of host microbe interactions. Several pathogens have evolved ways to expose sialic acid on their surface and hence to escape complement killing and opsonization by mimicry. Sialic acids are incorporated into capsules by *E. coli* K1 [23], Group B *Streptococci* [24], Serogroups B, C, W135 and Y *Neisseria meningitidis* [25]. The lipooligosaccharide of *Neisseria gonorrhoeae, Neisseria meningitidis* and *Haemophilus influenzae* are also sialylated [26]. In this case, a bacterial sialyltransferase uses CMP-Neu5Ac from the host as a substrate [26]. Sialic acids can also be synthesized from lactate by *Neisseria* itself, demonstrating a close link between metabolism and evasion of innate immune defenses [27].

Besides molecular mimicry, many microbes can utilize sialic acids as a source of carbon and nitrogen like *E. coli* K1, *H. influenzae* or *C. perfringens* [28]. Their metabolism comprises a permease for uptake and a neuraminiate lyase for conversion to N-acetyl mannosamine, which is either degraded or used in sialic acid biosynthesis. A number of commensal and pathogenic bacteria are also endowed with a sialidase, a glycosylhydrolase that cleaves sialic acid from sialo-glycoconjugates. Bacterial sialidases have been thought since a long time to contribute to virulence in bacteria that colonize mucosal surfaces such as *Vibrio cholerae, Streptococcus pneumoniae,* group B *streptococci*, *C. perfringens* and *B. fragilis* but the exact role of sialidase on virulence remains controversial [29]. Recently, it was shown that a sialidase is involved in the formation of *Pseudomonas aeruginosa* biofilms and hence contributes to colonization of the lungs during the initial stages of infection in cystic fibrosis patients [30]. In *S. pneumoniae,* a sialidase initiates an extensive deglycosylation of different host proteins, including IgA1 and human secretory component [31]. Furthermore, the sequential action of exoglycosidases sustains growth of *S. pneumoniae* on human α-1 acid glycoprotein, though growth is not as robust as on sucrose and lactose. Although the genetic analysis suggests that sugars from the glycan chain would sustain growth, this has not been shown directly [32].

In the present study, which is among the very first on the pathogenesis mechanisms of *C. canimorsus,* we demonstrate that a sialidase allows *C. canimorsus* to feed on glycan chains from glycoproteins. The role of sialidase is not to supply sialic acid since growth of the sialidase-deficient mutant could not be restored by adding sialic acid to the culture medium. Thus, we have a situation similar to that of *S. pneumoniae*: the role of sialidase is to provide access to masked sugars of surface-exposed glycoproteins. Growth of the sialidase-deficient mutant could be restored by amino sugars like GalNAc, GlcNAc and LacNAc but not by glucose, galactose, mannose or sialyl-lactose, indicating that the nutritional requirements of *C. canimorsus* are very different from those of *S. pneumoniae.*

Our study thus confirms the importance of a sialidase to initiate a deglycosylation process for bacterial metabolism. Moreover, in comparison with *S. pneumoniae, C. canimorsus* uses sialidase to feed on glycoproteins exposed at the surface of epithelial cells or even of macrophages, in spite of the fact that they do not adhere to these cells. The observation of extracellular bacteria specifically feeding on the surface of epithelial cells is not unprecedented. It has been described for *B. thetaiotaomicron*, a major commensal from the intestine, which feeds on fucosylated intestinal cells. Colonization by *B. thetaiotaomicron* even triggers the appearance of fucosyltransferase and fucosylated glycan expression [33]. Recent studies showed that host acquired fucose is incorporated by *B. fragilis* into capsular polysaccharide or glycoproteins, which in turn provides a survival advantage in the mammalian intestinal ecosystem [34]. As for *C. canimorsus*, it is likely that the capacity to feed on HeLa cells reflects the adaptation to feed on buccal epithelial cells.

Sialidase, which is pivotal in this feeding process, is surface localized and this surface localization is a prerequisite for unmasking glycan structures. It is not common to find enzymes anchored into the outer membrane, facing the outside of Gram-negative bacteria but there are examples like pullulanase a 116-kDa isoamylase of *Klebsiella oxytoca* [35]. Not surprisingly, SiaC is endowed with an N-terminal signal sequence, which turned out to be critical for its targeting. Sialidase thus crosses the cytoplasmic membrane via the Sec pathway but we have at present no explanation on how it crosses the outer membrane and remains anchored. It is probably not by a *C. canimorsus* specific mechanism since sialidase appeared to be also surface-exposed when expressed in *E. coli* (unpublished data). Sialidase could be a lipoprotein, like pullulanase. Alternatively, sialidase could be a surface-anchored auto-transporter protein like the *Y. enterocolitica* YadA [36]. However, the fact that the C-terminus of sialidase is not involved in the surface localization (unpublished data) argues against this hypothesis. Work in progress tries to address the question of how sialidase is anchored in the outer membrane.

Unlike what is observed with pullulanase, our data indicate that extremely little sialidase is released from *C. canimorsus.* This observation is in perfect agreement with the fact that *C. canimorsus* needs to be in direct contact with cells to feed on them. It also makes sense in the context of the mouth commensal microflora. Indeed, the oral cavity is occupied by some 500 different bacterial strains [37],[38], creating a fierce competition for nutrition. The fact that *C. canimorsus* does not release this enzyme suggests that *C. canimorsus* maximizes the benefit of sialidase by not sharing this fitness factor with competing bacteria. In agreement with this hypothesis, there is no cross-feeding when wt and *siaC Cc5* bacteria are inoculated together in the presence of macrophages. This implies that wt *C. canimorsus* must be extremely efficient in capturing the aminosugars that it extracts from the surface of cells and we hypothesize that *C. canimorsus* has dedicated high affinity transporters for these in its outer membrane.

Extracellular *C. canimorsus* replicated very efficiently not only when they were in direct contact with HeLa cells but also with J774.1 macrophages. Thus, *C. canimorsus* not only resists phagocytosis by cultured macrophages [13],[14], but they even take advantage of macrophages whose normal function is to engulf and kill microbes. To our knowledge, this is the very first report of a pathogen that can feed on phagocytic cells. This observation suggests that sialidase could contribute to virulence. We used a mouse tissue cage model in which the readout is bacterial persistence and we observed a dramatic difference in persistence between wt and sialidase-deficient *C. canimorsus*. Even more, we gained evidence that *in vivo, C. canimorsus* also feeds on phagocytes. These observations confirm our hypothesis that sialidase contributes to virulence, at least in the mouse model. It seems reasonable to extrapolate that it also plays a critical role during human infections. We would however be reluctant to call sialidase a virulence factor since it most probably evolved as a fitness factor for commensalism in the dog's mouth. Nevertheless, the mouse experiment shows that it may become a persistence factor if *C. canimorsus* is introduced in the tissues from another host. Our study thus shows once again the link between metabolism and virulence, as already well documented in studies on *Salmonella* [39], *Listeria* [40] and *Neisseria* [27]. However, unlike what was seen with *Salmonella*, there seem to be no or very little redundancy in the *in vivo* metabolism of *C. canimorsus* since the loss of sialidase had dramatic consequences on growth. It is interesting to observe that nutrition *in vivo* may be quite specific in spite of a very rich nutritional environment. Indeed, only GlcNAc and GalNAc could rescue growth while glucose had no effect and galactose was even deleterious. This difference could result from the fact that unlike *Salmonella*, *C. canimorsus* is a commensal highly adapted to its niche and only exceptionally a pathogen. Specialization is probably the hallmark of a bacterium that is primarily a commensal and only rarely a pathogen. Finally, *C. canimorsus* represents one more example illustrating that the distinction between commensals and pathogens is illusive. Commensalism and pathogenesis are two faces of the same coin.

Influenza neuraminidases have been successfully targeted with chemotherapeutic inhibitors for prophylaxis and treatment [41]. Given the wide prevalence and important role of sialidases in microbial infections, inhibition of bacterial sialidases could also provide a mechanism to prevent bacterial spreading during infections. Here, we observed a significant inhibition of the growth of *C. canimorsus* in the presence of macrophages by Neu5Ac2en. These preliminary data indicate that microbial sialidases could indeed serve as an attractive drug target to prevent bacterial dissemination.

## Materials and Methods

### Bacterial strains and growth conditions

*C. canimorsus 5* was routinely grown on Heart Infusion Agar (HIA; Difco) supplemented with 5% sheep blood (Oxoid) for 2 days at 37°C in presence of 5% CO_2_. Bacteria were harvested by gently scraping colonies off the agar surface, washed and resuspended in PBS. *C. canimorsus* was also grown in Heart Infusion Broth (Difco) supplemented with 10% (v/v) fetal bovine serum (FBS; Invitrogen) for approximately 24 h without shaking in an 37°C incubator with 5% CO_2_. Selective agents were added at the following concentrations: erythromycin, 10 µg/ml; cefoxitin, 10 µg/ml; gentamicin, 20 µg/ml; ampicillin, 100 µg/ml.

### Cell Culture and Infection

Murine monocyte-macrophage J774A.1 cells (ATCC TIB-67) were cultured in RPMI 1640 (Invitrogen) supplemented with 10% (v/v) FBS (Invitrogen), 2 mM L-glutamine and 1 mM sodium pyruvate. Human epithelial HeLa cells (ATCC CCL-2) and canine epithelial MDCK kidney cells (ATCC CCL-34) were grown in DMEM (Invitrogen) with 10% (v/v) FBS. Cells were seeded in medium without antibiotics at a density of 10^5^/cm^2^ and cultured at 37°C in humidified atmosphere containing 5% CO_2_. Unless otherwise indicated, infection was performed after 15 h at a moi of 20 representing 2×10^6^ bacteria per ml in each well at 37°C.

Monosaccharides and disaccharides (Sigma Aldrich) were added to 0.1% (w/v) final concentration. Neu5Ac and CMP- Neu5Ac were added to 0.01% final concentration.

*Cc5* was pretreated with 1mM Neu5Ac2en at 37°C for 30 min. Subsequently, infection of J774.1 was carried out in presence of 1 mM Neu5Ac2en during 24 h.

### Arbitrarily Primed PCR

Primers specific to the ends of the transposon and primers of random sequence that may anneal to chromosomal DNA sequences in close proximity to the transposon insertions were used in two rounds of PCR before sequencing. The first round of amplification was carried out in 50 µl containing 100 ng of genomic DNA, 1.5 mM MgCl_2_, 200 µM of primers 5′ CAGAATTCTGTTGCATTTGCAAGTTG 3′ complementary to Tn*4351* and 5′ggccacgcgtcgactagtacNNNNNNNNNNacgcc3′, 2.5 U of DNA polymerase (DyNAzymeII, Finnzymes), 200 µM of each dNTP, in 10 mM Tris HCl (pH 8.3) for 6 cycles (94°C for 1 min, 30°C for 1 min, 72°C for 2 min) and 30 cycles (94°C for 1 min, 45°C for 1 min, 72°C for 2 min) and final 10 min at 72°C. 10 µl of PCR product containing random fragments was used as template in a second round of 30 cycles of amplification (94°C for 30 sec, 45°C for 30 sec, 72°C for 1 min) using primers 5′ CAGAATTCTGTTGCATTTGCAAGTTG 3′ and 5′ GGCCACGCGTCGACTAGTAC 3′, from the 5′ of the random primer. PCR products were purified using NucleoSpin® from Machery Nagel. 20- 50 ng of random sized products were sequenced using an ABI sequencer. The Tn integration site was further confirmed by using primers on chromosomal DNA by sequencing towards the Tn integration site. Primers used were 5′ AATTGTTGTAACGATTGTCG 3′ or 5′ GCGAAGCGTTATCCCAAAGC 3′ complementary to the *siaC* sequence in a sequencing reaction containing 2 µg genomic DNA of *siaC*, betaine 0.25 M and BigDye Terminator Ready Reaction (PE Biosystems) with an initial denaturation step for 5 min and subsequent 99 cycles (95°C for 30 sec, 50°C for 20 sec, 60°C for 4 min).

### RNA isolation and reverse transcription (RT) PCR

*Cc5* were grown for 2 days on HIA blood plates. RNA was isolated from 5×10^8^ bacteria by a hot phenol/chloroform extraction method followed by DNase I (Amersham Pharmacia) treatment (0.5 U/µg RNA) for 2 h at 37°C. RNA was further cleaned by using a RNeasy kit (Quiagen) and stored at −80°C until use. An additional DNase I digest was introduced with 0.25 U/µg RNA for 15 min at 37°C and stopped by addition of final 2.5 mM EDTA and heat inactivation at 75°C for 10 min. Subsequent reverse transcription was performed with 50 U Superscript II/µg RNA in RT buffer (Invitrogen), 10 mM DTT and 50 µM specific primer (5129: 5′ GGGTAATCCGCACTTGTCGGG3′ or 5132: 5′ GTTTAGTTCTTGATAAATTCC 3′) for 60 min at 42°C and stopped at 70°C for 10 min. 10% of cDNA preparation or of a preparation made without addition of reverse transcriptase was subjected to PCR using following primer combinations: 4130 (5′ GGGTAACAACAAAAACCACTG 3′)+5129; 4132 (5′ TATAAGAATAATTGGTGGGC 3′)+5129; 4130+5132. 100 ng of genomic DNA from *Cc5* was used as a positive control of the PCR reactions.

### Construction of complementation and expression plasmids

Full length *siaC* was amplified with 5′ CATACCATGGGAAATCGAATTTTTTATCTT 3′ and 5′ GTTCTAGAGAGTTCTTGATAAATTCCTCAACTG 3′ primers and cloned into the *E. coli- C. canimorsus* shuttle vector pMM47.A [16] with *Nco*I and *Xba*I, leading to the insertion of a glycine at position 2 and a C- terminal histidine 6× tag in plasmid pMM52 (*siaC*_FL_). Forward primer 5′ AAAGCCATGGGAAACGTAATCGGCGGAGGCG 3′ was used with the same reverse primer to construct pMM50 (*siaC*_Δ1–21_), deleting the first 63 bp of *siaC*, but still including methionine and glycine at position 1 and 2, respectively, and using a C-terminal His 6× tag. The catalytic mutation in *siaC* of was introduced by site directed mutagenesis with an inverse PCR on pMM52, using primers 5′ GAAGGATTTGGGTGTTCGTGTATGTCG 3′ and 5′ CGACATACACGAACACCCAAATCCTTC 3′ leading to pMM59 (*siaC*_Y488C_). Plasmids derived from pMM47.A contained the *cfxA* gene originating from *Bacteroides sp.* and could be selected in *C. canimorsus* with 10 µg/ml cefoxitin [16]. The beta-lactamase also present on pMM47.A was used as a selection marker in *E. coli*.

The cDNAs encoding SiaC_Δ1–21_ (pHS2) were subsequently amplified using 5′ GGAATTCCATATGAACGTAATCGGCGGAGGC 3′ plus 5′ CGCGGATCCCTAGTTCTTGATAAATTCCTC 3′ and cloned into the expression vector pET15b(+) (Novagen). Plasmid pHS3 encoding SiaC_Δ1–21,Y488C_ was constructed by site directed mutagenesis on template pHS2 using the same primers as described for pMM59. All constructs were sequenced with an ABI sequencer. The sequence of SiaC was deposited at GenBank (accession number: EU329392).

### Purification of recombinant SiaC and immunoblotting

Expression of *siaC* constructs in *E. coli* BL21(DE3) was induced with 0.5 mM isopropyl-β-D-1-thiogalactopyranoside at A_600_ = 0.5 for 3 h. Proteins were purified by affinity chromatography using chelating Sepharose (Pharmacia) charged with NiSO_4_ according to the manufacturer's instructions. Samples were analyzed by SDS-PAGE by the system of Laemmli, and immunoblotted. Polyclonal serum from rabbit was generated against recombinant SiaC_Δ1–21_. The antigen was injected at days 0, 14, 28, and 56 with a final bleeding at day 80 (Laboratoire d'Hormonologie, Marloie, Belgium).

### MUAN hydrolysis

10^7^ bacteria were incubated with 0.006% 2′-(4-Methylumbelliferyl)-α-D-N-acetylneuraminic acid (MUAN) in 0.25 M sodium acetate pH 7.5 at 37°C for 3 min. Reactions were stopped with 50 mM Na_2_CO_3_ pH 9.6 and fluorescence was determined at 445 nm with a Wallac Victor^2^ 1420 Multilabel counter (Perkin Elmer).

### Outer Membrane Preparation

Bacterial cells resuspended in PBS containing DNase and RNase (10 µg/ml), were sonicated on ice. Unbroken cells were removed at 3000× g for 15 min, and total membranes were collected at 20 000× g for 30 min at 4°C. The membranes were suspended in PBS and sarcosyl (N-Lauroylsarcosine sodium salt, Sigma) was added to a final concentration of 1% (v/v). After incubation on ice for 1 h, membranes were collected at 20 000× *g* for 30 min and resuspended in electrophoresis sample buffer and analyzed by SDS-PAGE by the system of Laemmli.

### Immunofluorescence of bacteria

10^7^ bacteria were incubated on poly-D-lysine (BD) coated glass slides for 1 h at 37°C and subsequently fixed with 3% paraformaldehyde for 15 min. Anti- SiaC polyclonal serum (1∶500) and a FITC conjugated secondary antibody (Goat Anti- Rabbit IgG, Southern Biotech) was used at 1 µg/ml and fluorescence was measured with a Leica DMIRE2 microscope. Pictures were taken with a digital camera (Hamamatsu Photonics) and analyzed with OpenLab software (version 3.1.2) and Adobe Photoshop CS3 (version 10.0.1).

### Lectin Staining

10^5^ J774.1 macrophages or HeLa epithelial cells were seeded on poly-D-lysine coated slides. Infection was carried out with 4×10^7^ bacteria for 2 h. Uninfected cells were alternatively treated with purified recombinant SiaC at 100 ng/ml. Cells were fixed with 3% paraformaldehyde for 15 min. Biotinylated lectins SNA and PNA (Vector Laboratories) were incubated with cells at 2 µg/ml and 2.5 µg/ml, respectively, for 1 h. After washing with PBS, cells were treated with 1 µg/ml fluorescein conjugated streptavidin (Vector Laboratories) and fluorescence was determined on mounted slides (Vectashield, Vector Laboratories).

### Mice and tissue cage infection model

12 week-old male C57BL/6 mice were maintained under pathogen-free conditions in the Animal Facility of the Department of Research, University Hospital Basel. Animal experiments were performed in accordance with the guidelines of the Swiss veterinary law. Teflon tissue cages were implanted subcutaneously in the back of anesthetized mice as previously described [19]. The cages consisted of closed Teflon cylinders (10 mm diameter, 30 mm length, internal volume 1.84 ml) with 130 regularly spaced 0.2 mm holes. 2 weeks after surgery, 200 µl of bacterial suspension was injected percutaneously into the cage. Prior to infection, sterility of the tissue cage was verified. Tissue cage fluid (TCF) was sampled at day 2, 5, 9, 14 and 27 and examined for leukocytes and bacterial viable counts. Leukocytes from TCF were quantified with a Coulter counter (Coulter Electronics) and differentiated by Diff-Quick (Medion Diagnostics) Wright staining of cytospins and examined under light microscopy. The percentage of viable leukocytes was assessed by trypan blue exclusion.

The survival of *siaC* bacteria in the competition experiment was compared directly with wt *Cc5* in individual animals giving a 1∶1 ratio of wt to mutant bacteria. The number of mutant (erythromycin resistant) and wt bacteria recovered from the TCF of animals was established by plating to media with and without erythromycin. The competitive index was calculated as the (number of mutant/wild-type bacteria recovered from animals)/(number of mutant/wild-type bacteria in the inoculum).

### Statistical analysis

For growth experiments, means and standard deviation (s.d.) were calculated and statistical significance was evaluated by using a two- tailed, unpaired Student's *t* test. Differences were determined to be significant when p<0.05. For *in vivo* experiments, individual mouse values are shown including the median value of each group. Mann Whitney test with the *post hoc* Bonferroni correction was used for comparison between *Cc5* and *siaC* cfu numbers during infection.
