# Correction: *Capnocytophaga canimorsus*: A Human Pathogen Feeding at the Surface of Epithelial Cells and Phagocytes

**DOI:** 10.1371/annotation/8d781d6d-5d4e-46ea-afd9-bb3ede03fcb4

**Published:** 2008-10-22

**Authors:** Manuela Mally, Hwain Shin, Cécile Paroz, Regine Landmann, Guy R. Cornelis

In the Results section, Neu5Ac stands for N-Acetylneuraminic acid (not N-Acetyl-2,3-dehydro-2-deoxyneuraminic acid).

